# Factors that inhibit reporting of child maltreatment among dental health personnel – a scoping review

**DOI:** 10.1186/s12903-025-07287-2

**Published:** 2025-12-16

**Authors:** Eline Hokstad, Anca Virtej, Ingfrid Vaksdal Brattabø

**Affiliations:** 1 Department of Clinical dentistry, University of Bergen, Årstadveien 19, Bergen, 5009 Norway; 2 Department of Clinical Dentistry, Faculty of Health Sciences UiT, The Artic University of Norway, Hansine Hansens Veg 86, Tromsø, 9019 Norway; 3 Oral Health Centre of Expertise in Western Norway, Postboks 2354, Bergen, Møllendal 5867 Norway

**Keywords:** Child maltreatment, Child abuse, Child neglect, Dental health personnel, Failure to report, Obligation to report, Reporting barriers, Child protection, Child welfare services, Scoping review

## Abstract

**Background:**

Child maltreatment impacts the social, physiological, and psychological well-being of affected children, resulting in both short- and long-term consequences. Despite the obligation of dental health personnel to report suspected cases to child welfare services (CWS) or other appropriate authorities, various barriers hinder reporting, leading to underreporting. This scoping review aims to explore the factors that inhibit reporting of child maltreatment among dental health personnel by addressing the following research questions:

- 1. Which factors inhibit reporting of child maltreatment among dental health personnel?
- 2. To what degree do dental health personnel fail to report suspected cases of child maltreatment to CWS?

**Methods:**

A scoping review was conducted following the Preferred Reporting Items for Systematic Reviews and Meta-Analyses extension for Scoping Reviews (PRISMA-ScR) checklist. Studies were retrieved by performing systematic searches of PubMed, Embase (Ovid), SveMed+, Idunn and The Norwegian Dental Journal (Den norske tannlegeforenings Tidende) for articles published from 2013 to October 2024.

**Results:**

Of 303 identified articles, 34 were included in this review. Identified barriers to reporting child maltreatment among dental health personnel included diagnostic uncertainty, lack of knowledge, and fear of consequences. Targeted education and training programs were shown to improve reporting behaviors. However, overall reporting rates remain low, while the prevalence of failure to report suspected cases of child maltreatment was consistently high across the reviewed studies. These findings underscore the need for continued efforts to address the barriers to report.

**Conclusion:**

This study highlights key barriers faced by dental health personnel in reporting child maltreatment, such as diagnostic uncertainty, lack of knowledge, and fear of consequences. Alarmingly, a significant portion of dental health personnel fail to report suspected cases, perpetuating underreporting. This failure not only delays critical intervention but also allows the maltreatment to continue, compounding harm to vulnerable children. Addressing these reporting barriers is imperative to ensure that dental health professionals are equipped to fulfill their safeguarding role effectively. Future research should focus on closing knowledge gaps, expanding geographical representation, and standardizing reporting protocols to create a more robust and responsive reporting system.

**Trial registration:**

The project protocol for this review was preregistered on OSF, accessible via the following registration DOI 10.17605/OSF.IO/RBG2H.

**Supplementary Information:**

The online version contains supplementary material available at 10.1186/s12903-025-07287-2.

## Background

### Child maltreatment

Child maltreatment impacts the exposed children’s social, physiological, and psychological function and has both short- and long-term consequences for the affected children [1, 2]. Therefore, child maltreatment is a severe societal problem.

The definition of child maltreatment varies slightly depending on country and society. In the present review, the definition used for child maltreatment is based on the definition from World Health Organization (WHO) [3, 4]:

*“Child abuse or maltreatment constitutes all forms of physical and/or emotional ill-treatment*,* sexual abuse*,* neglect or negligent treatment or commercial or other exploitation*,* resulting in actual or potential harm to the child’s health*,* survival*,* development or dignity in the context of a relationship of responsibility*,* trust or power.’’ * [3]*.*

Definitions of the different forms of child maltreatment used in this article are derived from the Lancet series of Child Maltreatment by Gilbert et al. [2], presented in Table 1 below.

**Table 1:** Definitions of the different forms of child maltreatment

Types of child maltreatment	Definition
Physical abuse	Intentional use of physical force or implements against a child that results in, or has the potential to result in, physical injury.
Sexual abuse	Any completed or attempted sexual act, sexual contact, or non-contact sexual interaction with a child by a caregiver
Psychological or emotional abuse	Intentional behavior that conveys to a child that he/she is worthless, flawed, unloved, unwanted, endangered, or valued only in meeting another’s needs.
Neglect	Failure to meet a child’s basic physical, emotional, medical/dental, or educational needs; failure to provide adequate nutrition, hygiene, or shelter; or failure to ensure a child’s safety
Witness to intimate partner violence	Any incident of threatening behavior, violence, or abuse (psychological, physical, sexual, financial, or emotional) between adults who are, or have been, intimate partners or family members, irrespective of sex or sexuality

Definitions are derived from the Lancet series on child maltreatment by Gilbert et al. [2], with some modifications

### Consequences of child maltreatment

Numerous studies have investigated the global prevalence and extent of child maltreatment. Global estimates indicate that physical abuse affects 4% to 16% of children, while approximately 10% of children in high-income countries experience neglect or emotional abuse [2]. Self-report studies provide further insight, estimating prevalence rates of 12,7% for sexual abuse (7,6% among boys and 18% among girls), 22,6% for physical abuse, 36,3% for emotional abuse, 16,3% for physical neglect, and 18,5% for emotional neglect [5]. These studies indicate variations in prevalence rates across different studies [2, 5]. According to Gilbert et al. 80% of child maltreatment is committed by parents or parental guardians. In contrast, sexual abuse is predominantly perpetrated by acquaintances or other relatives [2]. However, many cases of child maltreatment against children go unreported, resulting in significant underestimation [2, 5–8].

Child maltreatment affects children´s development and can have various consequences. Since maltreated children often experience multiple forms of abuse, it is challenging to pinpoint specific consequences for each type of maltreatment. However, the impact of child maltreatment is often severe, lifelong, and sometimes fatal [1, 5]. Adverse childhood experiences increase the risk of mental and physical health problems, as well as behavioral issues, aggression, violence, and criminality. Victims of child maltreatment are at higher risk of developing mental health conditions such as post-traumatic stress disorder, depression, self-injurious behavior, or attempted suicide. Besides the previously mentioned mental health issues, they are at an increased risk of developing alcohol and drug abuse, along with various lifestyle-related problems [1, 2, 9–12]. They may also experience physical health problems like chronic pain or obesity, as well as behavioral and mental health challenges, including risky sexual behavior, which increases the risk of sexually transmitted infections [1]. Additionally, there is a higher risk of low educational achievement, low-skilled employment, and other difficulties in adapting to and contributing to society [1, 2]. Therefore, child maltreatment, with its severe consequences, is a major public health and social welfare issue that must be addressed and actively worked against.

### Regulating laws

The United Nations Convention on the Rights of the Child (UNCRC) is a legally-binding international agreement between the United Nations which is meant to secure equal rights and growing up circumstances for all children, regardless of ethnicity, social status, politics, religion and other influencing factors on a child´s childhood [13]. Within the context of child maltreatment article 19, article 34 and article 39 of the UNCRC is particularly significant [14]. These articles state that children have the right to be protected from all forms of violence, sexual abuse, and exploitation. They also specify that children who have been vulnerable and victimized are entitled to physical and psychological recovery, as well as social reintegration. To prevent and identify abuse, neglect, and exploitation, and to support those who are victimized, the articles require governments to establish laws and systems [14].

Guided by the UNCRC as the overarching legislation, dental health personnel worldwide are obligated to report suspected child maltreatment to the authorities. However, the specific laws governing this duty differs across countries, cultures, and legal systems. In Norway the key regulations for dental health personnel include the Child Welfare Act, the Health Personnel Act and the Dental Health Service Act [15–17]. The Norwegian Health Personnel Act Chap. 1, Sect. 33 mandates that health personnel must report any suspicions of child maltreatment or serious neglect to the children’s welfare service, irrespective of confidentiality obligations. Additionally, they are required to provide information upon request from the relevant authorities [15]. In most countries, it is mandatory to report child abuse and neglect (CAN) to the authorities. Nevertheless, in some countries, such as the United Kingdom (UK), health professionals are not mandated to report concerns about CAN but are required to do so under government guidance and as an ethical duty [18, 19]. Further guidance is issued by healthcare professional societies and by leading children’s organizations like the National Society for the Prevention of Cruelty to Children. The General Dental Council guidelines state that all registered dental professionals in the UK must be familiar with local procedures, know whom to contact for advice, and understand how to refer suspected cases to the appropriate authority, regardless of whether they work for the National Health Service or in private practice. Consequently, dental health personnel in the UK have a duty to report suspicions if a child is in a harmful situation [20–22]. In Australia, dentists are mandated to report CAN in four out of eight jurisdictions [21]. The different legal requirements in different countries or jurisdictions significantly influence how dental practitioners respond to CAN cases, likely leading to differences in understanding child protection guidelines and affecting factors that inhibit reporting [21].

Despite legal requirements and obligation to report, scientific literature indicates that dental health personnel underreport suspected cases of child maltreatment [3, 7, 23]. This underreporting poses a significant challenge in dental healthcare today, as it delays or hinders the detection of child maltreatment. Early detection is essential to minimize the severe short- and long-term consequences for the child and to ensure prompt removal from the abusive environment [1, 2, 8]. To promote early detection of child maltreatment, it is essential to gain a deeper understanding of the factors that inhibit reporting of child maltreatment among dental health personnel.

### Aims of the scoping review

The primary aim of this study was to clarify the available research in the field and enhance our understanding of dental health personnel as reporters – specifically the extent to which they fail to report suspected cases of child maltreatment and the factors that hinder reporting. Previous research has identified a persistent gap between suspicion and action, along with a need for improved training. To address this, it is essential to understand the magnitude of the challenges, map the barriers that prevent reporting, and identify which barriers are most common and critical to address. Given the diversity in study designs and methodologies, a scoping review was chosen to capture the breadth of existing evidence and provide a comprehensive overview of the topic. This review addresses the following research questions:

1. Which factors inhibit reporting of child maltreatment among dental health personnel?
2. To what degree do dental health personnel fail to report suspected cases of child maltreatment to CWS?

## Materials and methods

A scoping review was conducted to identify studies on factors that inhibit reporting of child maltreatment among dental health personnel. To ensure quality, the methodology was carried out and reported in accordance with the Preferred Reporting Items for Systematic Reviews and Meta-Analyses (PRISMA) guidelines [24, 25].

Open Science Framework (OSF), was utilized for the preregistration of the study [26]. The project protocol for this is accessible via the following registration DOI: 10.17605/OSF.IO/RBG2H.

### Identifying relevant studies

Studies were identified in the scientific databases PubMed, Embase/Ovid, SveMed + and Idunn. All searches were performed in collaboration with a librarian at the University of Bergen, Norway. The searches were conducted by using paired search terms with Boolean operators with the following keywords, “dentists”, “dental auxiliary”, “dental auxiliaries”, “dental health care worker”, “dental assistant”, “dental hygienist”, “dental technician”, “child maltreatment”, “child abuse”, “child mistreatment”, “child neglect” or “neglected child”, supervised by a research librarian. Additionally, the Norwegian terms “barnemishandling” and “tann” were used for searches conducted in Idunn. Mesh terms from PubMed were adapted for Embase/Ovid, SveMed + and Idunn. Each search incorporated relevant free text terms, truncated when necessary to capture alternative word endings. The search covered literature published from 2013 to October 2024.

To ensure comprehensive representation, as the SveMed + database, which indexes articles in Scandinavian languages, has not been updated since 2019 formal search strategy methods were combined with an additional search in The Norwegian Dental Journal (Den norske tannlegeforenings Tidende) to identify publications written in Scandinavian languages [27]. By including these recent publications, we ensure that relevant literature in Scandinavian languages published after 2019 is adequately represented.

The following is an example of one of the literature searches. This search was performed in Ovid/Embase in June 2023, in collaboration with research librarian at University of Bergen, Norway and yielded 134 articles.

1. exp dentist/29,524
2. exp dental auxiliary/10,985
3. (dental auxiliar* or dentists or dental health care worker* or dental assistant* or dental hygienist* or dental technician*).ti, ab, kf. 32,298
4. 1 or 2 or 3 55,460
5. exp child abuse/45,124
6. (child abuse or child maltreatment* or child mistreatment* or child neglect or neglected child*).ti, ab, kf. 19,657
7. 5 or 6 48,440
8. 4 and 7 411
9. limit 8 to yr="2013 -Current” 134

For full search strategy see Additional file 1.

### Selection criteria

To narrow the results from initial, main and additional searches the following inclusion- and exclusion criteria were set:

### Data acquisition

Identified articles were imported to Rayyan, a web-based screening tool [28]. Title and abstracts of each article were independently screened by two authors. After screening all articles’ separately, conflicted results were discussed and re-analyzed. Discussion with a third reviewer was undertaken to resolve any confusion and disagreements between the initial two reviewers. Studies that violated inclusion- and exclusion criteria were excluded (Tables 2 and 3). Included articles were read in full. Studies that did not meet the inclusion and exclusion criteria were excluded after full-text review. The main findings from the full-text articles were collected and compiled in Table 4 to provide a comprehensive overview.

**Table 2:** Inclusion criteria

1. Peer-reviewed publications
2. Children from 0–18 years
3. Dental health personnel working with children (0–18 years)
4. Papers in English, Norwegian, Swedish or Danish
5. Articles from 2013–2024
6. Includes one of these terms: Dentists, Dental Auxiliaries, Dental Auxiliar*, Dental auxiliary, Dental Health Care worker, dental assistant, dental hygienist, dental technician, child abuse, child maltreatment, child mistreatment, child neglect, neglected child, barnemishandling (Idunn/Norwegian), tann (Idunn/Norwegian)

**Table 3:** Exclusion criteria

1. Adults, defined as persons over 18 years old
2. Articles published before 2013
3. Case presentations including less than 5 cases
4. Descriptive reviews, commentaries, letters and conference abstracts
5. Articles published in other languages than English, Norwegian, Swedish or Danish

**Fig. 1:**
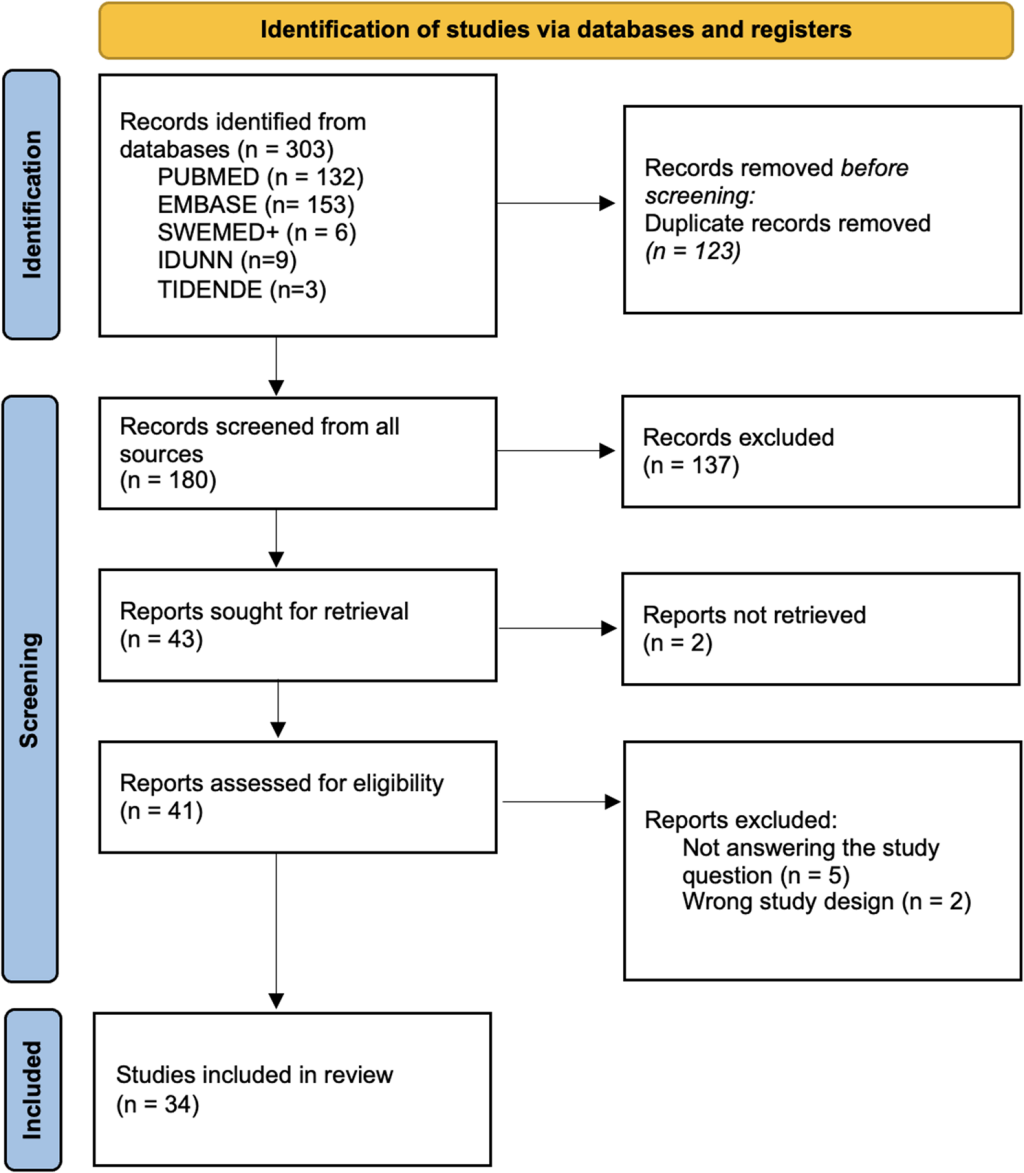
PRISMA flow diagram. Flow diagram of the screening and selection process adapted from the Preferred Reporting Items for Systematic Review and Meta-Analyses (PRISMA) statement

The respective data were presented for each study: Author´s name, year of publication, country, practitioners, study design, sample size (n)/response rate (%), suspected cases of child maltreatment and prevalence of reporting (%), prevalence of failure to report (%) and reasons for failure to report. The PRISMA 2020 flow diagram (Fig. 1) outlines the inclusion process for eligible studies as described by Page et al. [24]. The data from the included studies were compiled into Table 4. Following the methodological guidance for systematic scoping reviews, no critical appraisal of the quality of the studies included was performed [63].

## Results

### Study characteristics

Out of the 303 initially identified studies, 123 duplicates were removed before screening. The remaining 180 unique studies were screened by title and abstract using the Rayyan citation manager. This process identified 43 reports, of which two could not be retrieved in full text. Consequently, 41 reports were read in full text, and 34 were selected for inclusion (presented in Fig. 1).

The majority of the included studies were conducted in Asia (*n* = 14) and Europe (*n* = 15), with two studies each from Oceania and South America, and one from Africa. Paper publication years of the papers ranged from 2013 to October 2024, as determined by the inclusion and exclusion criteria outlined in Tables 2 and 3.

### Factors that inhibit reporting of child maltreatment among dental health personnel

The review identified several significant barriers that inhibit dental health personnel from reporting suspected cases of child maltreatment. Diagnostic uncertainty was one of the most frequently reported barriers, with practitioners often struggling to distinguish signs of maltreatment from symptoms of other medical conditions [29–33, 35, 38–40, 42, 44, 45, 47, 48, 52, 54, 55, 57–59, 61]. The extensive number of studies citing this issue underscores its prevalence. Lack of knowledge about child maltreatment and the reporting process was another major barrier, cited in many studies [29, 31–33, 35, 38, 40–47, 50–60]. This included limited awareness of the legal obligation to report and insufficient training on recognizing abuse indicators. Fear of consequences also emerged as a critical issue, with practitioners expressing concerns about potential harm to the child, backlash from caregivers, or legal repercussions for themselves [29–38, 40–43, 45–49, 52–55, 57, 59–61]. Additional barriers, such as mistrust of CWS and reluctance to get involved, were mentioned in several studies but were less frequently reported [37, 47, 49]. These findings highlight the multifaceted challenges faced by dental health personnel in fulfilling their reporting responsibilities.

### Prevalence of failure to report suspected cases of child maltreatment

The prevalence of failure to report suspected cases of child maltreatment among dental health personnel was found to be consistently high across the reviewed studies. For instance, Pawis et al. reported that 42% of the suspected cases went unreported [56]. Similarly, Al-Ahmad SH et al. reported that only 32% of dental practitioners reported cases, leaving 68% unreported [31]. Markovic N et al. found an even lower rate, with just 9% of suspected cases reported and 51% explicitly stating they would not report suspected cases [50].

Other studies corroborated these findings, revealing a persistent trend of low reporting rates and high failure frequencies [32, 33, 42, 49, 58, 62]. Regional variations were evident, with reporting rates generally higher in high-income countries. For example, Alapulli H et al., reported a referral rate of 51,3% among the participants in Finland, a high-income country, compared to Singh RK et al., who found that only 14,1% of practitioners in India, a middle-income country, took action [33, 58]. Despite these differences, the overall reporting frequency remained insufficient, even in countries with robust training programs. These findings indicate a significant gap between suspicion and reporting, highlighting the need for targeted interventions to address barriers and improve adherence to legal reporting obligations.

Table 4 summarizes the literature published on the frequency of reporting, failure to report and the factors that inhibit reporting of child maltreatment among dental health personnel, covering the period from 2013 to October 2024.

**Table 4:** Key characteristics of included studies and overview of their results (*n* = 34)

Author (year)	Country	Dental health personnel	Study design	Response rate (%)/ Sample size (*n*)	Suspected cases and prevalence of reporting (%)	Prevalence of failure to report (%)	Reasons for failure to report
Al Hajeri H et al. (2018) [29]	United Arab Emirates (UAE)	UAE Dentists	CSS	Response rate: 77% Sample size: *n* = 501	Most of the responders reported that they had never suspected child abuse in their practice, while 39.9% (*n* = 152) suspected such cases. Prevalence of Reporting: Unknown	Unknown	Lack of knowledge of referral process (60.2%) Fear of family violence (59.6%) Lack of diagnosis certainty (54.9%) Fear of the consequences to the child from the intervention of the statutory agencies (48%) Fear of litigation (37.7%) Fear of family violence to you (28.2%) Concerns about impact on the practice (financial time taken, loss of income, income withdrawal) (28.1%)
Al Hashmi R et al. (2021) [30]	Dubai and Northern United Emirates	Dental assistants (DAs) and Dental hygienists (DHs)	CSS	Response rate: 62% Sample size: *n* = 186 (*n* = 137 dental assistants and 49 dental hygienists)	Unknown	Unknown	Afraid to get in trouble with the parent (49.1%) Did not know to whom to report (43.1%) Afraid of wrong diagnosis (32%) Worried reporting would lead to further violence (26.9%) Afraid dentist won’t accept interference (23%) Worried about getting in trouble with authorities (18.9%) Did not know how to take history about injury (11.5%)
Al-Amad SH et al. (2016) [31]	United Arab Emirates (UAE)	Dentists working in various clinics in the public and private sectors in the seven emirates of the UAE.	CSS	Response rate: 55% Sample size: *n* = 193	47 participants (25%) have encountered a suspicious case of child abuse, and only 15 of them (32%) reported it. *This means that only 7.8% of all respondents have reported a case of child abuse.*	Of the 47 participants suspecting a child abuse case, 32 of them (68%) did not report it. *Corresponding to that 16.5% of the total sample size failed to report child abuse cases.*	I was afraid that my diagnosis of abuse is wrong (32%) I did not know to whom to report (21%) I did not know how to take history about the injury (17%) I was afraid I would get in trouble with parents (16%) I was worried that reporting a suspicious case would lead to further violence (13%) I was worried about losing the patient and his/her family (2%) I was worried about getting in trouble with authorities (1%)
Al-Dabaan R et al. (2014) [32]	Saudi Arabia	Saudi Arabia Dental Society	EQ	Response rate: Unknown Sample size: *n* = 122	59% reported that they have suspected cases of CAN, but only 7% of dentists contacted social services and 2.8% contacted the police. *This means that 5.7% of the total sample contacted the social services or the police.*	19.7% of the dentists with CAN cases in their practice did not take any action. *Corresponding to that 11.5% of the total sample size failed to report child abuse and neglect.*	Fear of violence toward the child (87.7%) Lack of certainty about the diagnosis (79.8%) Lack of knowledge of referral procedures (78.9%) Fear of negative effects on the child´s family (58.8%) Fear of negative impact on dental practice Fear of litigation
Alapulli H et al. (2023) [33]	Finland	Dentists (D), dental hygienists, and dental nurses (DNs) in Finland	EQ	Response rate: 18.7% Sample size: *n* = 1586	43% of the respondents have suspected child abuse. 51.3% of these have made a referral to the social services. *This means that 22% of all respondents have ever made a referral to CWS.*	Of the participants suspecting child abuse 64.3% did not report it. *Corresponding to that 27.6% of all respondents have failed to report CAN cases.*	Uncertainty about the observation (D = 80.7%, DNs = 79.1%) Lack of knowledge regarding procedures for referral (D = 42.9%, DNs = 45.7%) Fear of violence to the child (D = 33.4%, DNs = 35.3%) Fear of violence to you (D = 22.9%, DNs = 21.3%) Fear of the consequences to the child, if the authorities intervene (D = 20.2%, DNs = 22.5%) Fear of litigation (D = 16.9%, DNs = 24.7%) Others (D = 13.5%, DNs = 11.5%) Concerns about impact on the practice (D = 5.2%, DNs = 6.1%)
Alapulli H et al. (2024) [34]	Finland	Finnish dental professionals	Q	2008: Response rate: 32.4% Sample size: *n* = 625 2019: Response rate: 18.7% Sample size: *n* = 1025	2008: Unknown 2019: 515 (50.3%) of the respondents have suspected physical abuse, and 275 (26.9%) of all respondents had made a referral to CWS in the 2019 survey.	2008: Unknown 2019: The self-reported number of non-reporters to the CWS or the police was 327 (32.1%).	Negative consequences to the child at home: 2008: (44.5%), 2019: (56.4%) Worried about getting in trouble themselves: 2008: (30.2%), 2019: (36.3%)
Bjørknes R et al. (2019) [35]	Norway	Public dental health personnel in Norway (Dentists (D) and Dental Hygienists (DHs))	CSS	Response rate: 77.8% Sample size: *n* = 1200 This study is based on responses from 366 dental hygienists and dentists who reported reasons for not reporting suspected child maltreatment to the CWS.	Unknown	366 of the participants reported that during their careers, they had suspected child maltreatment or neglect that they had not reported to the CWS. *Corresponding to that 30*,*5% of the total sample size failed to report child maltreatment.*	Was unsure of own assessment (90.4%) Did not have enough knowledge about child abuse and neglect (68%) Was not sure how one would document findings/suspicion (64.2%) Unpleasant to report since one cannot be anonymous (60.7%) Was afraid of parental reaction (53%) Had no procedures for sending reports to CWS (48.9%) Was afraid of what would happen to the family (45.1%) Was afraid of what could happen to the child (41.6%) Was afraid that the child would stop going to the dental clinic (38.4%) Was afraid of how CWS would address report of concern (35.6%) Was afraid of what would happen to the parents (29.9%) Was afraid of getting threats (26.4%) Had no one with whom to discuss the concern (26%) Was unsure how to send a message of concern (22.3%) Was unsure about where the message of concern should be sent (22.3%) Was afraid for the reputation of the dental clinic in the community (13.3%) Was bound by confidentiality (11.6%) Lacked support form leader of the clinic (10.7%)
Borgmo EØ et al. (2024) [36]	Norway	Public dental health personnel in Norway (Dentists (D) and Dental Hygienists (DHs))	EQ	Response rate: 70.9% Sample size: *n* = 1270 This study is based on responses from 389 participants who reported that they did not meet their obligation to report suspected cases.	Unknown	389 of the participants reported that they did not meet their obligation to report suspected cases. 32,9% of the respondents did not report suspected child maltreatment to the CWS.	Was unsure of own assessment (D = 86.4%, DHs = 86.0) Fear of mistakenly reporting (D = 77.3%, DHs = 70.2%) Was afraid of parental reaction (D = 53.6%, DHs = 56.1%) Unpleasant to report since one cannot be anonymous (D = 53.6%, DHs = 54.4%) Did not have enough knowledge about child abuse and neglect (D = 36.4%, DHs = 40.4%) Was not sure how one would document findings/suspicion (D = 40.0%, DHs = 28.1%) Was afraid that the child would stop going to the dental clinic (D = 38.2%, DHs = 29.8%) Was afraid of what would happen to the family (D = 36.4%, DHs = 29.8%) Was afraid of getting threats (D = 32.7%, DHs = 33.3%) Was afraid of what would happen to the child (D = 31.8%, DHs = 28.1%) Was afraid of what would happen to the parents (D = 20.9%, DHs = 17.5%) Was afraid of how CWS would address report of concern (D = 27.3%, DHs = 15.8%) Was afraid for the reputation of the dental clinic in the community (D = 11.8%, DHs = 7.0%) Was unsure how to send a message of concern (D = 16.4%, DHs = 10.5%) Was unsure about where the message of concern should be sent (D = 10.0%, DHs = 1.8%) Had no procedures for sending reports to CWS (D = 12.7%, DHs = 14.0%) Lacked support from leader of the clinic (D = 8.2%, DHs = 10.5%) Lacked support from colleagues (D = 10.9%, DHs = 14.0%) Had no one with whom to discuss the concern (D = 16.4%, DHs = 5.3%) Was bound by confidentiality (D = 6.0%, DHs = 12.3%)
Buldur B et al. (2022) [37]	Turkey	Dentists in Turkey	CSS	Response rate = 100% Sample size: *n* = 229	Unknown	Unknown	Fear that the child would be harmed (30.4%) Insufficient evidence to file a report (26.8%) Afraid that parents will hurt the dentist (25.74%) Mistrust to child protection services (23.58%)
Cukovic-Bagic I et al. (2015) [38]	Croatia	Dentists registered in the Croatian Chamber of Dental Medicine (CCDM) in 5 major Croatian cities (Zagreb, Varaždin, Osijek, Rijeka, and Split).	Q	Response rate: 93.75% Sample size: *n* = 510	A total of 134 (26.27%) respondents reported to have had suspicion of child abuse or neglect on one or more occasions during their professional career. 21 (4.1%) participants reported suspicion within the last 6 months. Of those 9 (42.9%) respondents had referred the cases to the social services or police. *This means that only 1.8% of the respondents have reported to the social services or police.*	Unknown	Fear of family violence towards child Uncertainty about observations Fear of consequences to the child due to authorities’ intervention Lack of knowledge regarding referral procedure Fear of violence towards own family Concerns about impact on practice Other
da Silva RA et al. (2013) [39]	Brazil	Dentists in Sao Luis	CSS	Response rate: 60.2% Sample size: *n* = 301	31.3% of the dentists reported recognizing child abuse within the last 5 years. Among respondents who suspected child abuse, 84% reported it to the relevant authorities. *This means that 26.2% of all respondents have made a report of concern.*	16% of the dentists who suspected child abuse did not report suspected child abuse cases to the authorities. *Corresponding to that 5% of the total sample size have not made a report of concern to the relevant authorities.*	Fear of litigation and its impact on practice (33.3%) Uncertainty of the relevant authorities (26.7%) Uncertainty of the diagnosis (20%) Lack of evidence (13.3%) Prefer to talk with parents first (6.1%)
Dimitrova, M. M et al. (2021) [40]	Bulgaria	Dental practitioners from several regions of Bulgaria	Q	Response rate: Unknown Sample size: *n* = 265	A total of 60 (22.3%) respondents reported having suspected cases of CAN, and 5.7% of them had reported the case to the authorities.	Unknown	Lack of knowledge of the way or procedures of reporting (40.4%) Fear of subsequent violence against the child (23.4%) Unsure in the diagnosis of CAN (14.9%) Fear of consequences for the child when the relevant institutions interfere (12.8%) Fear of aggression against the dentist (6.4%) Fear of a subsequent trial (2.1%)
Gupta S et al. (2023) [41]	India	Dental professionals practicing in India	CSS	Response rate: Unknown Sample size: *n* = 422	Unknown	Unknown	Lack of adequate knowledge and awareness about the role of dental professionals (71%, *n* = 300) Lack of adequate knowledge in identifying (53.8%, *n* = 227) Lack of knowledge of reporting procedures (52.1%, *n* = 220) Fear of negative impact on dental practice (27.5%, *n* = 116) Fear of litigation (25.1%, *n* = 106) Presence of parents/family members (15.9%, *n* = 67) Others (0.5%, *n* = 2)
Harris CM et al. (2013) [42]	Scotland	General Dental practitioners (GDPs) in Scotland	Q	Response rate = 52% Sample size: *n* = 628	37% (*n* = 235) of respondents had suspected child abuse/neglect in one or more of their paediatric patients but only 11% (*n* = 72) of all respondents had referred a case.	17% (*n* = 107) of the dentists admitted they had suspected a case of child abuse/neglect but had not reported it when directly questioned about this issue.	Lack of certainty of the diagnosis 74% (*n* = 465) Fear of violence to child 52% (*n* = 324) Fear of consequences to child from statutory agencies 46% (*n* = 286) Lack of knowledge of referral procedures 43% (*n* = 271) Fear of litigation 35% (*n* = 220) Fear of violence to GDP 31% (*n* = 195) Concerns of impact on practice 6% (*n* = 38)
Harris JC et al. (2022) [43]	UK	Paediatric dentists (members in British Society of Paediatric Dentistry)	CSS	2005: Response rate: 66.3% Sample size: *n* = 448 2016: Response rate: 62.4% Sample size: *n* = 295	2005: 67.9% suspected, 30.7% referred. 2016: 82.3% suspected, 61% referred.	2005: 37.2% have suspected maltreatment but never referred. 2016: 21.3% have suspected maltreatment but never referred.	Lack of certainty about diagnosis: 2005: 77.7% (*n* = 334), 2016: 52.9% (*n* = 153) Fear of family violence to child: 2005: 53.0% (*n* = 222), 2016: 58.8% (*n* = 171) Fear of consequences to child from statutory intervention: 2005: 53.7% (*n* = 225), 2016: 33.8% (*n* = 97) Concerns about confidentiality: 2005: 35.6% (*n* = 148), 2016: 16.4% (*n* = 47) Fear of family violence to self: 2005: 31.8% (*n* = 130), 2016: 27.5% (*n* = 79) Lack of knowledge of referral procedures: 2005: 30.2% (*n* = 124), 11.8% (*n* = 34) Fear of litigation: 2005: 29.1% (*n* = 120), 2016: 22.0% (*n* = 63)
Hussein AS et al. (2016) [44]	Malaysia	Paediatric dental specialists (PDs), general dental practitioner (GDPs), dental nurses (DNs) and Paediatric dentistry postgraduate students (PGs) who attended a National Paediatric Dentistry Conference in, Malaysia	Q	Response rate: 74.7% Sample size: *n* = 108 (PDs = 37, PGs = 3, GDPs = 30, DNs = 38.) Since PGs were only three, they were included in GDPs group (PDs = 37, GDPs = 33,DNs = 38)	Only a few of the participants stated that they have reported abuse cases (PDs = 24.3%, GDPs = 3.0%, DNs = 15.8%)	Unknown	Lack of adequate history (PDs = 48.6%, GDPs = 45.5%, DNs = 36.8%) Uncertainty about the diagnosis of abuse (PDs = 29.7%, GDPs = 48.5%, DNs = 34.2%). Not sure who to report to (PDs = 21,6%, GDPs = 33,3%, DNs = 31,6%) Lack of certainty regarding whether reporting is legal (PDs = 2.7%, GDPs = 3.0%, DNs = 23.7%) Did not want to get involved (PDs = 13.5%, GDPs = 15.2%, DNs = 28.9%) Lack of knowledge about consequences of abuse (PDs = 13.5%, GDPs = 21.2%, DNs = 18.4%) Others (PDs = 10.8%, GDPs = 3.0%, DNs = 0%)
Jakobsen U et al. (2019) [45]	Faroe Islands	Dental professionals (dentists and dental hygenists) in Faroe Islands	Q	Response rate: 72% Sample size: *n* = 51 (*n* = 33 dentists and *n* = 18 dental hygienists)	31 (61%) of the respondents experienced suspicion of child maltreatment at some point during their career. 39% (12/31) had given notice of their suspicion in at least one case. *This means that 23.5% of all respondents have reported child maltreatment to the authorities during their career.*	71% (22/31) of the respondents who had suspected child maltreatment during their career did not notify anyone of their concern. *This corresponds to that 43% of all respondents had failed to report child maltreatment cases.*	Uncertainty as to whether the suspicion was reliable (90%) Fear of the consequences for the child (31%) Lack of procedural knowledge (27%)
Kaur H et al. (2017) [46]	India	Dental professionals in India	CSS	Response rate: 96.7% Sample size: *n* = 1914	Unknown	Unknown	Lack of knowledge about the CA and dentist’s role in reporting (51.4%) Lack of adequate history (34.6%) Concern about its effect on their practice (14%)
Kural D et al. (2020) [47]	Turkey	Members in Turkish Dental Association (TDA)	Q	Response rate = 5.1% Sample size: *n* = 1020	17.1% of the participating dentists reported having suspected cases of CAN the last 5 years, but only 1% reported the cases.	Unknown	Not being able to obtain the patient’s history to report (45%) Worrying about the child being further abused (18.8%) Not having known about the legal responsibility of reporting (18.3%) Mistrust of the child protection service agencies (9.4%) Anticipating being harmed by the child´s family (4.2%) Not wanting to report and get involved (2.5%) Not having the required time to report the case (1.2%) Thinking that the child´s family would be harmed (0.7%)
Kugananthan S et al. (2021) [48]	Australia	Dental Health Professionals (DHPs)	EQ	Response rate: 7% of DHPs in Western Australia Sample size: *n* = 228 (*n* = 78 general dentists, *n* = 29 specialists/specialist registrars, *n* = 76 dental hygienists and *n* = 46 oral health therapists)	Unknown	Unknown	Uncertainty of diagnosing abuse (81%) Potential legal ramifications (58%) Concern for the child´s safety (55%) Unsure of proper reporting protocol Concern for own safety
Laud A et al. (2013) [49]	Greece	Dentists registered with the Dental Associations of Athens and Piraeus	Q	Response rate = 83.6% Sample size: *n* = 368	18% of dentists that suspected CM in their young patients kept records of their observations. Only 6 of the 368 respondents (1.6%) made an official report of a suspected case of CM.	35% of respondents suspected CM but preferred not to report it.	Doubt over diagnosis (44%) Fear of consequences for the child (20%) Nothing would prevent me (18%) Do not know (17%) Unaware of agency responsible (17%) Involvement in legal proceedings (9%) Consequences to my profession (3%) Possible threats of violence (3%) Other (2%)
Markovic N et al. (2015) [50]	Bosnia and Herzegovina	Dentists working in 7 different towns in Bosnia and Herzegovina	CSS	Response rate: 70% Sample size: *n* = 210	If suspected CAN, only 9% of the respondents (*n* = 18) would report it.	51% of the respondents (*n* = 107) answered that they would not report the case if they suspected a CAN case	Lack of knowledge and the procedure of reporting (43%) Never had a case, lack of knowledge about procedure of reporting (31%) Lack of adequate history (15%) No answer (6%) Lack of confidence that reports will be correctly investigated and fear that it may cause more harm than good (5.1%)
Martins-Júnior PA et al. (2019) [51]	Brazil	Dentists (D), physicians (P) and nurses (N) employed in public services in Diamantina/MG	Q	Response rate: Unknown Sample size: *n* = 62 in total (27 dentists, 10 physicians and 25 nurses)	7.4% (*n* = 2) of the dentists, 90% (*n* = 9) of the physicians and 28% (*n* = 7) of the nurses participating reported suspected cases of physical abuse.	Unknown	Lack of knowledge (D = 40.7%, *P* = 100%, *N* = 68%) Do not know where to make the complaint (D = 7.4%, *P* = 90%, *N* = 28%) Insecurity in misreporting (D = 33.3%, *P* = 40%, *N* = 12%) Professional negligence (D = 0%, *P* = 60%, *N* = 16%) Fear presented by professionals (D = 51.9%, *P* = 60%, *N* = 52%) Non-resolution of cases by competent authorities (D = 0%, *P* = 30%, *N* = 4%)
Mogaddam M et al. (2016) [52]	Saudi Arabia	Pediatric dentists, pediatric dentistry residents (masters, PhD, and Saudi Board students), and dental interns practicing in all of the dental schools in Jeddah	CSS	Response rate: 77% Sample size: *n* = 208	22 participants (11%) had suspected a case of child abuse in their clinics and only 6 (3%) of them reported the cases.	Of the 22 participants suspecting a child abuse case, only one (4.5%) did not take any action.	Lack of knowledge about referral procedures (60%) Fear of anger from family members and parents (27%) Uncertainty about the diagnosis of the case as child abuse (21%) Possible harmful effect on the child from the family (21%) Lack of adequate history about the case (14%)
Olatosi OO et al. (2018) [53]	Nigeria	Dental residents (Dentists attending a postgraduate update course)	Q	Response rate: Unknown Sample size: *n* = 179	Only 22 respondents (25.9%) document signs of suspected cases of CAN in patient’s records, 12 (14.1%) contacted the social welfare and 3 (3.5%) contacted the police.	No action taken when suspecting a case: *n* = 48 (56.5%) *Corresponding to that 26.8% of the respondents have failed to report their concern.*	Lack of knowledge in referral procedures (65.4%) Concerns about confidentiality (59.2%) Fear of consequences to the child (57.5%) Lack of knowledge in diagnosis of CAN Fear of negative effect on child’s family Fear of violence against dentist No legal obligation to reporting CAN Reporting CAN is against my social norms Unavailable social service in my institution
Özgür N et al. (2020) [54]	Turkey	Paediatric dentists in Turkey	EQ	Response rate: 40.9% Sample size: *n* = 212	43.9% suspected physical abuse: however, only 12.7% (*n* = 27) reported it.	Unknown	I did not know where and how to report (38.5%) Lack of the documents for reporting (36.9%) The concern about possible further injury to the child (29.2%) The fear of anger from patient relatives (26.2%) Being unsure (21.5%)
Patrick A et al. (2020) [55]	United Kingdom (UK)	Health care practitioners, nurses, dental practioners and doctors within Surrey and Sussex Healthcare National Health Service Trust (SASH)	Q	Response rate: Sample size: *n* = 276 Group 1: *n* = 100 Group 2: *n* = 100 Group 3: *n* = 76	Experience of reporting to a child protection team was 64% (*n* = 64) in Group 1, 43% (*n* = 43) in Group 2 and 51% (*n* = 38) in Group 3.	Unknown	Unsure of the diagnosis Concerns for the safety of the child and the professionals themselves Concerns for consequences from CPT Lack of knowledge of reporting procedure Fear for legal ramifications
Pawis et al. (2022) [56]	Germany	Dentists throughout Germany	EQ	Response rate: Unknown Sample size: *n* = 264	Suspected cases: *n* = 319, how many dentists had suspected CAN? Prevalence of reporting: Unknown	*n* = 134 (42%) of the suspected cases were not referred (?)d	Uncertainty to who to contact in the event of suspected cases (34.9%) Uncertainty of the diagnosis (31.8%) Not enough knowledge about child abuse (30.3%) Unsure how to report suspicion (28.4%) Concern if the suspected case turns to be unfounded (25.8%)
Saleem MN et al. (2021) [57]	Pakistan	Dental practitioners across Pakistan	CCS	Response rate: 82.5% Sample size: *n* = 330	Only 20% of participating dentists had ever suspected an individual as physically abused. Out of those who suspected, only 30% reported it to legal authorities. *This means that only 6% of the total sample have made a report to legal authorities.*	Unknown	Fear of anger (59%) Possible harmful effects (54%) Lack of knowledge (47%) Uncertain Diagnosis (24%) Lack of history (19%)
Singh, R. K. et al. (2023) [58]	India	Dentists working in government and private hospitals in the Varanasi district, Uttar Pradesh State, India	CSS	Response rate: 90.7% Sample size: *n* = 611	Only 86 (14.08%) out of 611 practitioners had acted on the suspected CAN cases.	525 (85.92%) participants reported that they did not take any action in suspected CAN cases.	Fear of anger from patients and family (23%) Possible effect on child´s family (19%) Lack of adequate history (18%) Possible effect on my practice (14%) Uncertainty of diagnosis (12%) Lack of knowledge of referral procedures (10%) Fear of litigation (2%) No reason (2%)
Soumya Mohanan TV et al. (2020) [59]	India	Dental practitioners in Belagavi City, India	CSS	Response rate: 92.7% Sample size: *n* = 102	Unknown	Unknown	Lack of knowledge in referral procedure (45%) Consequences to the child (15.7%) Concern about confidentiality (9.8%) Lack of certainty in diagnosis (8.8%) Fear of negative impact on dental practice (6.9%) Fear of litigation (5.9%)
Tilvawala D et al. (2014) [60]	New Zealand	Dental therapists	Q	Response rate = 49.8% Sample size: *n* = 320	12.5% of the respondents suspecting child maltreatment said they would refer the case to a paediatric dentist while 37.6% said they would directly notify Child Youth and Family Services (CYFS)	Unknown	Fear of mistakenly reporting a non-abuse case (68.6%) Lack of history Lack of knowledge about child maltreatment Fear of further family violence towards child Lack of knowledge about reporting protocols Limited confidence in child protection services Do not want to confront family Fear of losing patients No time with a busy practice schedule
Uldum B et al. (2017) [61]	Denmark	Dentists and dental hygienists in Denmark	Q	Response rate: 67% Sample size: *n* = 964	Of the 40.8% respondents who had had suspicion CAN at some point during their career, 50.0% had referred their concern to social services	During their career, 30.0% of the respondents reported having had a suspicion without referring to social services.	Uncertainty about the observations, signs and symptoms of abuse and neglect (72.4%) Fear of additional violence towards the child when the caregiver learns about the referral (56.5%) Uncertainty about the referral procedures (53.7%) Fear of the consequences to the child, if the authorities intervene (50.4%) Others Fear of litigation Violence towards self Potential impact on practice
van Dam BAFM et al. (2015) [62]	The Netherlands	General dental practitioners (GDP) in the Netherlands	EQ	Response rate: 25% Sample size: *n* = 264	When suspecting domestic violence or child abuse most GDP’s (81%) made a note of their suspicion and/or acted in these cases (58%), but only 18% reported the case.	10% of the respondents asked about a recent case answered that they neither made a note in the patient’s record, nor t other actions. *Corresponding to that only 2.3% of the total sample have made a report.*	Unsure about whether their suspicions were correct (88%)

*Q* Questionnaire, *EQ* Electronic Questionnaire, *CSS* Cross-Sectional Study. The numbers in italic text under the prevalence of reporting and prevalence of failure to report in Table 4 are converted values generated by one of the researchers. This conversion was performed to enable easier comparison of the results and to obtain data reflective of the entire sample size, rather than only individuals suspecting child maltreatment

## Discussion

This scoping literature review sought to investigate and consolidate information on the factors that inhibit reporting of child maltreatment among dental health personnel and associated prevalence of underreporting. The following sections explore and discuss the findings and methodological aspects of the current study.

### Factors that inhibit reporting of child maltreatment among dental health personnel

Dental health personnel face several significant barriers to reporting suspected cases of child maltreatment, which hinder early intervention and effective support for victims [1, 2]. The main barriers identified in this review include uncertainty of diagnosis, inadequate patient history, lack of knowledge – either about child maltreatment or the reporting procedure itself – and fear of consequences for the child or negative repercussions for the dental practitioner. Additional factors were mistrust of CWS, reluctance to get involved, and fear of trouble with authorities (see Table 4).

#### Diagnostic uncertainty and lack of knowledge

Diagnostic uncertainty arises when signs of maltreatment are subtle or overlap with other conditions, making it challenging for practitioners to confidently identify abuse [64, 65]. In some cases, inadequate patient history also contributes to diagnostic uncertainty. When relevant background information is missing or incomplete – such as previous injuries, familiy context, or behavioral indicators – dental professionals may struggle to interpret clinical signs in a broader safeguarding context. This issue is compounded by insufficient knowledge of child maltreatment and inadequate training on reporting procedures, leaving some practitioners unable to recognize key signs and feeling unprepared to act [64–66]. Dental health personnel may hesitate to report due to uncertainties in their own assessments, insufficient knowledge of reporting procedures or unclear understanding of applicable laws. Furthermore, each child and concern is unique, which adds complexity and underscores the lack of definitive answers in such cases. This issue is not unique to dentistry. For example, Martins-Júnior et al. highlighted that physicians and nurses face similar barriers, including limited knowledge, uncertainty about where to report, fear of misreporting, unresolved cases by authorities, and even professional negligence [51]. Such findings emphasize that dental health personnel are a part of a broader challenge shared across disciplines. To address these barriers, it is essential to increase awareness, develop supportive policies, provide targeted education and facilitate multidisciplinary collaboration. Studies have shown that targeted training improves practitioners’ ability to recognize signs of maltreatment and increases their confidence in reporting procedures [42, 67]. Supportive policies – such as clearer guidelines, legal protection, and structured referral pathways – can reduce uncertainty and fear of repercussions [38, 68]. Multidisciplinary collaboration, including regular meeting between dental services and child welfare teams, has been found to foster trust and improve communication, thereby enhancing reporting practices [69, 70]. While improving knowledge and diagnostic confidence is essential, other emotional and systemic factors also play a critical role in shaping reporting behavior. These efforts can empower dental professionals and other professionals to fulfill their critical role in safeguarding children more effectively.

#### Fear of consequences for the child and negative repercussions for the dental practitioner

Fear of the potential consequences for both the child and the dental practitioner is a significant deterrent to reporting suspected cases of maltreatment. Dental professionals may hesitate to act on suspicious abuse due to concerns about further harm to the child or facing personal and professional repercussions. A notable portion of included studies identified fear of consequences to the child as a significant barrier to reporting [29–38, 40–42, 45, 47, 49, 52–54, 58, 59, 61]. In contrast, fewer studies highlighted concerns about career-related consequences, such as potential legal or professional risks, as barriers [29, 32, 35, 38, 40, 41, 46, 48, 49, 53, 58]. The prevalence of fear for the child’s welfare was notably higher than fears related to the practitioner’s professional practice or career. This focus on the child is understandable and ethically grounded, as the child´s welfare should remain the central concern in child protection efforts. However, this concern may paradoxically contribute to hesitation if practitioners fear that reporting could lead to unintended harm – such as family disruption or inadequate follow-up by authorities.

For instance, Laud et al. found that 20% of practitioners cited fear of consequences for the child as a barrier, while only 3% reported concerns about career repercussions or potential violence against the dentist [49]. These emotional dilemmas highlight the need for clear communication, trust in child welfare systems, and reassurance that reporting is a protective act rather than a punitive one. Addressing these concerns – both for the child and for the practitioner’s career – is essential to overcoming barriers and ensuring effective reporting practices.

#### Mistrust of child welfare services, reluctance to get involved and fear of trouble with authorities

Mistrust of CWS, coupled with reluctance to become involved in potentially complex legal and social issues, represents another significant barrier to reporting suspected maltreatment. Dental practitioners may doubt the effectiveness or fairness of child protection systems, fearing that reports might not lead to meaningful interventions or could even exacerbate the child’s situation [35, 37, 47, 55, 60]. This mistrust may stem from various factors, including limited understanding of how CWS operates, negative portrayals in the media, personal experiences with CWS, or differing professional experiences across regions.

Collaboration between dental health personnel and child protection teams also varies by country or region. For example, in Norway, collaboration agreements and annual meetings between Public Dental Health Services (PDHS) and CWS aim to improve communication, promote mutual understanding, and share knowledge about each other’s roles [69–71]. Such initiatives can reduce fear of consequences and build trust in the system. In Norway, an increase in reporting by public dental health personnel was observed when it became mandatory for the PDHS to submit figures for reports to CWS to the municipal –state –reporting system KOSTRA [72, 73]. This further underscores the importance of promoting awareness within clinics, leadership and colleague networks.

Despite these tendencies towards better collaboration between dental health personnel and the CWS in recent years, there is still a need for further efforts to strengthen interdisciplinary cooperation, particularly in regions where such agreements are nascent or inconsistent. Additionally, some dental practitioners express hesitation to involve themselves in situations that could lead to prolonged interactions with authorities or entangle them in legal proceedings [30, 31, 33, 40, 41, 47–49, 55, 58, 59, 61]. Concerns about being drawn into court cases or facing scrutiny from law enforcement and regulatory bodies can deter practitioners from acting, especially if they feel unprepared for such scenarios. The fear of making an incorrect diagnosis further compounds this reluctance [29–31, 44, 48, 49, 51–53, 55, 56].

These challenges – including mistrust of CWS, reluctance to engage with legal processes, and fear of making incorrect assessments – highlight the complex emotional and systemic barriers that dental professionals face when considering whether to report suspected maltreatment. Addressing these concerns requires more than individual awareness: it calls for structural support through clear and accessible reporting procedures, legal protection, and emotional reassurance. By fostering trust in child welfare systems and strengthening interdisciplinary collaboration, practitioners can be empowered with the knowledge, confidence, and resources needed to act responsibly and effectively in safeguarding children.

### Prevalence of failure to report suspected cases of child maltreatment

The failure to report suspected cases of child maltreatment remains a critical concern, with substantial proportions of cases going underreported despite dental health personnel’s legal obligation to report. While reporting and failure-to-report rates provide useful indicators, they also reflect deeper issues related to professional confidence, perceived responsibility, and systematic support.

For instance, Al-Amad SH et al. found that out of 47 participants who encountered a suspicious case of child abuse, only 32% reported their suspicion, while 68% did not report it [31]. Similarly, Markovic N et al. found that only 9% of respondents would report suspected child maltreatment and neglect, while 51% would not report it [50]. These findings underscore a persistent gap between suspicion and action, suggesting that awareness of abuse does not automatically translate into reporting behavior. This disconnect may be influenced by factors such as fear of consequences, diagnostic uncertainty, and lack of trust in child welfare systems, as discussed in Sect. 4.1. Thus, the statistics not only quantify the problem, but also point to the need for more comprehensive support structures that address both knowledge and the emotional and institutional barriers to reporting.

Reporting and failure to report rates vary slightly across different studies, with a tendency for higher reporting frequencies in Western countries like Norway and Finland compared to non-Western countries like India, Turkey, and Saudi Arabia [68]. For example, Singh RK et al. reported that only 14,1% of participants acted on suspected CAN cases, while 85,9% did not take any action [58]. In contrast, Alapulli H et al.(2023) found that 51,3% of participants suspecting child abuse had made a referral to the social services, while 64,3% had on occasion not reported it [33]. This difference might reflect disparities in knowledge, training, and socio-economic factors, with middle-and low-income countries exhibiting larger knowledge gaps than high income countries. However, not all findings align with the general tendency of improved reporting rates with increased knowledge and training. For instance, Alapulli H et al. (2024), focusing specifically on suspected cases of child physical abuse, compared data from 2008 to 2019 and revealed a surprising decline in reporting rates despite enhanced knowledge and greater emphasis on child protection training [34]. The 2019 questionnaire revealed not only a lower response rate compared to 2008 but also reported higher percentages of barriers to reporting suspected cases. These findings suggest that educational and procedural advancements alone may not be sufficient, and that persistent emotional, cultural, or systemic factors can continue to hinder reporting behavior.

#### Reporting and barriers over time

Longitudinal comparative studies conducted within the same country suggest that reporting rates can improve over time, particularly when supported by targeted interventions, clearer procedures and increased training. For example, Cairns et al. (2005) used a questionnaire to examine the role of Scottish GDPs in child protection (CP) [74]. Building on this, Harris et al. (2010) explored changes over time in the gap between suspecting and referring cases of CAN, utilizing both Cairns et al.’s findings and their own adapted questionnaire [42, 74]. Harris et al. found that a higher percentage of respondents had received child protection training, during their undergraduate studies and pursued postgraduate training, more frequently in 2010 compared to earlier results by Cairns et al. (2005) [42, 74]. Furthermore, fewer Scottish dentists in 2010 admitted to suspecting cases of child abuse or neglect without reporting them when directly asked about this issue [42, 74]. A longitudinal study including 591 public dental health personnel in Norway revealed an improvement in reporting of suspected cases of child maltreatment from 59.6% in 2014 to 79.5% in 2019. However, corresponding figures for failure to report were 33.9% in 2014 and 37.9% in 2019 [68]. This simultaneous increase in both reporting and failure to report may reflect heightened awareness and diagnostic sensitivity, making practitioners more conscious of missed opportunities to act. Moreover, a 2024 Norwegian cross sectional study demonstrated an association between increased child protection training in recent years and a higher likelihood of filing reports of concern [67]. Although causation cannot be emphasized, these findings highlight the importance of child protection training. The findings from Norway with an increase in both reporting and failure to report over a 5-year period, may also indicate that increased knowledge makes dental personnel more aware and conscious of instances where they have failed to report.

Recent longitudinal data from the UK further supports these trends. Harris et al. (2022) conducted a repeated cross-sectional survey of paediatric dentists in 2005 and 2016, revealing substantial improvements in both training and reporting practices [43]. The proportion of respondents who had ever suspected child maltreatment increased from 67.9% to 82.3%, while those who had ever made a referral rose from 30.7% to 61.0%. Notably, the gap between suspicion and referral narrowed significantly, with the percentage of practitioners who had suspected but never referred declining from 37.2% to 21.3%. This study also identified key factors associated with reporting behavior, including specialist status, exposure to safeguarding procedures, and participation in multi-agency training. Despite these improvements, barriers such as diagnostic uncertainty, fear of consequences, and lack of procedural clarity remained influential, underscoring the need for continued structural and educational support [43].

Harris et al. concluded that while Scottish dentists were reporting more cases of CAN by 2010, similar barriers persisted [42]. This reinforces the notion that improvements in reporting behavior require not only educational efforts but also structural and emotional support mechanisms. Collectively, these studies indicate a positive trend, in which increased knowledge, training, and awareness of legislation correlate with higher reporting rates among dental health personnel [42, 67]. However, causality cannot be inferred from these findings, and the persistence of barriers underscores the need for multifaceted strategies to enhance practitioner responsiveness.

It is worth noting that a low prevalence of failure to report does not equate to a high prevalence of reporting, as overall reporting frequencies remain low. For instance, Mogaddam M et al. found that, while 11% of participants suspected child abuse, only 3% reported their suspicions [52]. These findings emphasize the need for continued efforts to address the barriers contributing to underreporting, particularly among dental health personnel. They also illustrate the disconnect between suspicion and action, which may be shaped by emotional stain, lack of confidence, or perceived lack of institutional support.

Knowledge about child maltreatment, including the ability to recognize its signs and understand the legal and procedural requirements for suspecting, detecting and reporting cases is essential in this context. As mentioned earlier, the global prevalence and extent of child maltreatment indicate that physical abuse affects 4% to 16% of children, while approximately 10% of children in high-income countries experience neglect or emotional abuse [2]. Furthermore, self-reported studies suggest even higher prevalence rates for various forms of abuse, highlighting discrepancies in findings across different studies [5]. Given this prevalence, dental health personnel are likely to encounter maltreated children or those at risk during routine examinations. This is particularly relevant in Nordic countries, where PDHS regularly see children aged 3–18 years [75]. Therefore, knowledge of child maltreatment, reporting procedures, and applicable laws is crucial for promptly identifying cases of child maltreatment and minimizing the negative consequences for victims [1, 2, 5, 7].

### Lack of knowledge and underreporting among dental health personnel

Limited knowledge about child maltreatment remains a central factor contributing to underreporting among dental health personnel [23]. Recognizing key signs, understanding what constitutes a concern, and knowing how to act are essential competencies in safeguarding practice [4, 64, 65]. Without this foundation, practitioners may overlook children at risk or hesitate to report due to uncertainty about procedures or legal obligations [7, 21, 22, 31]. Studies show that such uncertainty is linked to higher rates of non-reporting, which can delay intervention and prolong harm to vulnerable children [1, 2, 7]. Targeted training that addresses both clinical indicators and reporting protocols can reduce hesitation and strengthen practitioner responsiveness.

### Strengths and limitations

A key strength of this study is its comprehensive approach to developing search terms in collaboration with a research librarian and conducting new searches in October 2024 to ensure the inclusion of recent articles. Rigorous and transparent methods were employed throughout, including blind and independent data selection and extraction. The search strategy encompassed five electronic databases, with two independent reviewers assessing each title and abstract. A strength of this review is the use of the Rayyan screening tool, which facilitated transparent and collaborative decision-making during full-text screening, ensuring accurate inclusion of relevant studies. Additionally, this review included articles in both Scandinavian languages and English, enhancing the representation of Scandinavian research.

However, some limitations should be noted. Firstly, several articles lacked information on either the prevalence of reporting or failure to report, making it difficult to draw definitive conclusions. Secondly, there was an underrepresentation of studies from South America, Africa and Oceania compared to Europe and Asia, limiting the generalizability of findings to these regions. Thirdly, variability in study designs and definitions of reporting behaviors posed challenges for direct comparisons. Additionally, there are relatively few studies exploring barriers to reporting suspected child maltreatment, leaving limited foundational research to build upon.

Furthermore, despite a structured and multi-database search strategy, some relevant studies may have been missed due to limitations in keyword selection and database indexing. For example, studies using terms such as “oral health therapists” or published in journals not indexed in the selected databases where not retrieved in the initial search. This was the case for Han et al. (2022) and Fox et al. (2022), which were identified post hoc through reviewer feedback. These omissions highlight the importance of broader terminology and expanded database coverage in future reviews.

To strengthen future research, we recommend incorporating additional sources such as Scopus and Web of Science, and refining search terms to capture a wider range of professional titles and publication venues.

Further research should aim to address these gaps by including diverse geographical regions and standardizing definitions and methodologies, and refining search strategies to ensure comprehensive coverage of relevant literature.

## Conclusion

This scoping review identifies key barriers to reporting suspected child maltreatment among dental health personnel, including diagnostic uncertainty, insufficient knowledge, and fear of consequences. These factors contribute to underreporting and delaying critical interventions for vulnerable children.

Regional differences were observed, with higher reporting rates in high-income countries, likely reflecting disparities in training, legal frameworks, and systemic support. Longitudinal studies suggest that targeted education and clearer procedures can improve reporting behavior, yet overall rates remain low.

To strengthen child protection efforts, dental professionals must be equipped with the necessary knowledge, confidence, and institutional support. Future research should address knowledge gaps, expand geographical scope, and promote standardized reporting protocols to ensure timely and effective safeguarding practice.

## Supplementary Information

Supplementary Material 1.

## Data Availability

This scoping review does not involve the generation of new datasets. All data supporting the findings of the study are derived from previously published literature, which is fully cited within the manuscript. No new primary data were collected for this review.

## Abbreviations

CAN: Child abuse and neglect
CCDM: Croatian Chamber of Dental Medicine
CP: Child protection
CSS: Cross-Sectional Study
CYFS: Child Youth and Family Services
CWS: Child Welfare Services
D: Dentists
DAs: Dental Assistants
DHPs: Dental Health Professionals
DHs: Dental Hygienists
DNs: Dental Nurses
EQ: Electronic questionnaire
GDPs: General Dental Practitioners
OSF: Open Science Framework
P: Physicians
PDHS: Public Dental Health Services
PGs: Postgraduate students
PRISMA: Preferred Reporting Items for Systematic Reviews and Meta-Analyses
PRISMA-ScR: Preferred Reporting Items for Systematic Reviews and Meta-Analyses extension for Scoping Reviews
Q: Questionnaire
SASH: Surrey and Sussex Healthcare National Health Service Trust
UAE: United Arab Emirates
UK: United Kingdom
UNCRC: The United Nations Convention on the Rights of the Child
