# Supplementary material for: Factors that inhibit reporting of child maltreatment among dental health personnel – a scoping review
Source: BMC Oral Health. 2025 Dec 16;25:1911. doi: 10.1186/s12903-025-07287-2 (PMC12709845; doi:10.1186/s12903-025-07287-2)
Supplement: Supplementary file 1 — Supplementary Material 1. [file 12903_2025_7287_MOESM1_ESM.docx]

# Additional file 1

Literature searches were conducted with the help of a research librarian at the University of Bergen, covering literature from 2013 to October 2024. Most searches were conducted in May and June 2023, with additional searches in October 2024 to ensure the inclusion of recent literature. The additional file provides the full search strategy.

##

## Search string 1

Search string

Database PubMed, May 2023

(("Dentists"[MeSH Terms] OR "Dental Auxiliaries"[MeSH Terms] OR ("dental auxiliar*"[Title/Abstract] OR "Dentists"[Title/Abstract] OR "dental health care worker*"[Title/Abstract] OR "dental assistant*"[Title/Abstract] OR "dental hygienist*"[Title/Abstract] OR "dental technician*"[Title/Abstract])) AND ("Child Abuse"[MeSH Terms] OR ("Child Abuse"[Title/Abstract] OR "child maltreatment*"[Title/Abstract] OR "child mistreatment*"[Title/Abstract] OR "child neglect"[Title/Abstract] OR "neglected child*"[Title/Abstract]))) AND (2013:2023[pdat]

This search term gave us 111 articles when conducted in May 2023.

## Search string 2

Searches in Ovid/Embase conducted in June 2023, 134 articles:

1             exp dentist/       29524

2             exp dental auxiliary/       10985

3             (dental auxiliar* or dentists or dental health care worker* or dental assistant* or dental hygienist* or dental technician*).ti,ab,kf.      32298

4             1 or 2 or 3           55460

5             exp child abuse/              45124

6             (child abuse or child maltreatment* or child mistreatment* or child neglect or neglected child*).ti,ab,kf.                19657

7             5 or 6    48440

8             4 and 7 411

9             limit 8 to yr="2013 -Current"     134

Link to the performed search:

<https://ovidsp.ovid.com/ovidweb.cgi?T=JS&NEWS=N&PAGE=main&SHAREDSEARCHID=4HQ0DmtUL7yP1eu2MLymBu5H89eQD5S4wUgZfePOowEC0CPaTzmU1EYCVjFGZiJVm>

## Search string 3

Searches in SveMed+ conducted June 26^th^ 2023, yield 6 articles as shown below:


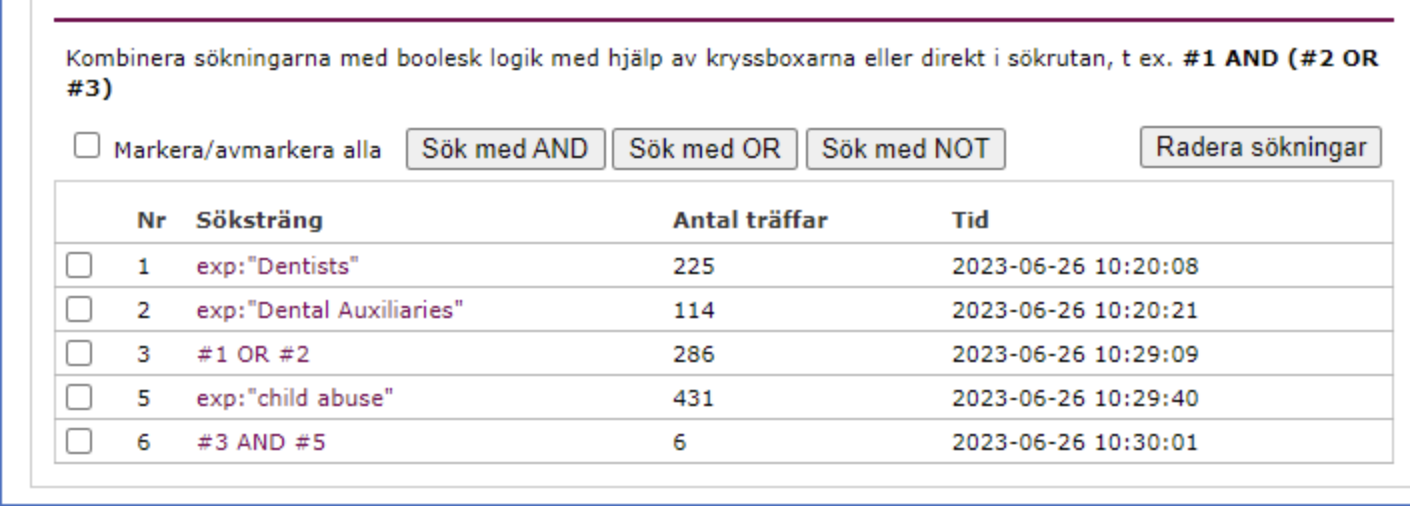


## Search string 4

In March 2024 there was done two searches in Idunn, one with Norwegian search terms and one with English search terms.

The Norwegian search string:

(Tann* AND barnemishandling) gives us 3 articles.

The following link shows the Norwegian search:

<https://www.idunn.no/action/doSearch?AllField=tann*+AND+barnemishandling&AfterYear=2013&BeforeYear=2021&queryID=51%2F32007234>

The English search string:

(Dentists* AND (child abuse OR child maltreatment)) gives us 4 articles.

The following link shows the English search:

<https://www.idunn.no/action/doSearch?AllField=Dentist*+AND+%28child+abuse+OR+child+maltreatment%29&AfterYear=2013&BeforeYear=2023&queryID=1/32007519>

## Search string 5

Search string 5 presents the new searches conducted in October 2024, distinguishing them from the initial searches in various databases (May 2023 in PubMed and June 2023 in Ovid) up to current search date.

New searches in Ovid were performed October 20^th^ 2024, and yields 16 new articles:

1     exp dentist/ (30848)
2     exp dental auxiliary/ (11264)
3     (dental auxiliar* or dentists or dental health care worker* or dental assistant* or dental hygienist* or dental
technician*).ti,ab,kf. (34247)
4     1 or 2 or 3 (58146)
5     exp child abuse/ (47544)
6     (child abuse or child maltreatment* or child mistreatment* or child neglect or neglected child*).ti,ab,kf. (20782)
7     5 or 6 (51129)
8     4 and 7 (428)
9     limit 8 to yr="2023 -Current" (16)

The following link shows the new search:

<https://ovidsp.ovid.com/ovidweb.cgi?T=JS&NEWS=N&PAGE=main&SHAREDSEARCHID=nMXGIydh6kLmmQQN1cRcZRSoD8Ye3qdP0yFNb5WDynhGQycETBhTnWvDK9CYu01E>

New searches in PubMed were conducted October 20^th^ 2024, and yields 21 new articles. Following search terms was repeated from the initial search:

(("Dentists"[MeSH Terms] OR "Dental Auxiliaries"[MeSH Terms] OR ("dental auxiliar*"[Title/Abstract] OR "Dentists"[Title/Abstract] OR "dental health care worker*"[Title/Abstract] OR "dental assistant*"[Title/Abstract] OR "dental hygienist*"[Title/Abstract] OR "dental technician*"[Title/Abstract])) AND ("Child Abuse"[MeSH Terms] OR ("Child Abuse"[Title/Abstract] OR "child maltreatment*"[Title/Abstract] OR "child mistreatment*"[Title/Abstract] OR "child neglect"[Title/Abstract] OR "neglected child*"[Title/Abstract]))) AND (2023:2024[pdat])

Both the Ovid search, which yielded 16 new articles, and the PubMed search, which yielded 21 articles, were imported to the screening tool Rayyan. Fourteen duplicates were detected and removed. The remaining 23 articles were read in full, and 2 of them were included in the review.

New English search in Idunn yield 2 new articles. Following link shows the new search:

<https://www.idunn.no/action/doSearch?AllField=Dentist*+AND+%28child+abuse+OR+child+maltreatment%29&AfterYear=2023&BeforeYear=2024&queryID=15/39783519>

Both these articles were excluded because they did not answer the study question. Therefore, this was not added to Rayyan.

The repeated search with the Norwegian search terms in Idunn did not provide any new articles.

All new searches go through the duplicate detection in the digital tool Rayyan.

## Search string 6

The additional search in The Norwegian Dental Journal (Den norske tannlegeforenings Tidende) was conducted October 20^th^ 2024 using the term “Barnemishandling”, yielding 35 articles, most of which were guidelines, which is not relevant in this context. The following link shows this search: <https://www.tannlegetidende.no/?q=barnemishandling> . Three more studies were retrieved and read in full text:

- Næss L et al. (2014): Tannhelsepersonellets rolle for å oppdage barn utsatt for mishandling eller omsorgssvikt.
  <https://www.tannlegetidende.no/journal/2014/11/d2e1561/Tannhelsepersonellets_rolle_for_å_oppdage_barn_utsatt_for_mishandling_eller_omsorgssvikt>
  This article was excluded because it does not answer the study question.
- Pedersen V et al. (2015): Tannhelsetjenestens rolle ved barnemishandling.
  <https://www.tannlegetidende.no/journal/2015/4/d2e259/Tannhelsetjenestens_rolle_ved_barnemishandling>
  This article was excluded because it does not answer the study question.
- Borgmo E et al. (2024): Hvorfor unnlater tannhelsepersonell å utdøve meldeplikten?
  <https://www.tannlegetidende.no/journal/2024/10/m-1611/Hvorfor_unnlater_tannhelsepersonell_å_utøve_meldeplikten>
  This article was included.

Of these only the article from Borgmo E et al. (2024) was included because the others did not meet the inclusion- or exclusion criteria.
